# A novel TGF-β receptor II mutation (I227T/N236D) promotes aggressive phenotype of oral squamous cell carcinoma via enhanced EGFR signaling

**DOI:** 10.1186/s12885-020-07669-5

**Published:** 2020-11-27

**Authors:** Hwa-Kyung Son, Dokyeong Kim, Yongwoon Lim, Jin Kim, Iha Park

**Affiliations:** 1 Department of Dental Hygiene, Yeungnam University College, Daegu, 42415 Republic of Korea; 2 Department of Dental Hygiene, Jeonju Kijeon College, Jeonju, 54989 Republic of Korea; 3 Department of Oral Pathology, Oral Cancer Research Institute, BK21 PLUS Project, Yonsei University College of Dentistry, Seoul, 03722 Republic of Korea; 4 Department of Biochemistry, Department of Biomedical Sciences, Research Institute of Medical Sciences, Chonnam National University Medical School, Hwasun, 58128 Republic of Korea; 5 Department of Biochemistry, Research Center for Aging and Geriatrics, Research Institute of Medical Sciences, Chonnam National University Medical School, Hwasun, 58128 Republic of Korea

**Keywords:** Transforming growth factor-β type II receptor, Oral squamous cell carcinoma, Epidermal growth factor receptor, Migration, Invasion

## Abstract

**Background:**

Transforming growth factor-β (TGF-β) signaling is a double-edged sword in cancer development and progression. TGF-β signaling plays a tumor suppressive role during the early stages of tumor development but promotes tumor progression in later stages. We have previously identified various mutations of TGF-β receptor II (TβRII) in human oral squamous cell carcinoma (OSCC) samples. In the present study we analyzed I227T/N236D mutation of TβRII, which was detected in the metastatic lymph node of an OSCC patient.

**Methods:**

The effect of I227T/N236D TβRII mutation on transcriptional activities was measured using DR26 cells, which lack functional TβRII. HSC2 human OSCC cells stably expressing wild-type and I227T/N236D mutant TβRII were generated and used to examine the effect of I227T/N236D TβRII mutation on xenograft tumor growth, in vitro cell proliferation, apoptosis, migration, and invasion.

**Results:**

The I227T/N236D mutation of TβRII upregulated TGF-β signaling and promoted xenograft tumor growth when compared with the wild-type, without affecting the in vitro proliferative capacities. To delineate the differences in proliferative capacities in vivo and in vitro, the apoptotic and survival signals were analyzed following curcumin treatment. Concomitant with apoptotic induction, epidermal growth factor receptor (EGFR) activation was observed upon curcumin treatment, which was further activated in I227T/N236D mutant transfectant cells when compared with wild-type cells. Enhanced EGFR activation correlated with cell survival and apoptotic resistance. Enhanced migratory and invasive capabilities of I227T/N236D mutant cells also depended on EGFR signaling.

**Conclusions:**

These results suggest that enhanced EGFR signaling via upregulated TGF-β signaling shifted the balance toward survival and promoted cell migration and invasion in I227T/N236D mutant cells, elucidating the role of I227T/N236D mutation of TβRII in OSCC progression.

## Background

Transforming growth factor-β (TGF-β) plays a critical role in biological processes, including development, homeostasis, fibrosis, and carcinogenesis [1–4]. TGF-β initiates signaling across the plasma membrane to the nucleus by binding to TGF-β type II receptor (TβRII) which, in turn, recruits TGF-β type I receptor [5, 6]. Following TGF-β receptor activation, receptor-associated Smad proteins (R-Smads), such as Smad2 and Smad3, are phosphorylated. Phosphorylated R-Smads recruit Smad4, and then, translocate to the nucleus to activate transcription of downstream target genes [4, 7, 8]. In addition to this canonical TGF-β signaling pathway, TGF-β also activates Smad-independent non-canonical pathways, including PI3K/AKT, mitogen-activated protein kinases (MAPKs), NF-κB, Rho/Rac1, Cdc42, FAK, Src, and Abl [4, 9]. TGF-β signaling is a double-edged sword in cancer development and progression. In the early stages of tumorigenesis, TGF-β functions as a tumor suppressor by inducing cell cycle arrest and apoptosis. Paradoxically, TGF-β signaling can exacerbate malignant phenotypes at later stages of tumorigenesis, by inducing epithelial-mesenchymal transition (EMT), tumor angiogenesis, and anti-immune reactions [4, 9–11]. Overexpression of TGF-β1 correlates with progression of carcinomas, including breast, colon, esophageal, gastric, lung, ovarian, and pancreatic cancers. In contrast, weak or no TGF-β signaling due to mutations of TβRII, have been found in gliomas, biliary, breast, colon, esophageal, gastric, lung, ovarian, esophageal, pancreatic, prostate, and head and neck cancers [7, 11, 12]. Oral squamous cell carcinoma (OSCC) represents one of the most common types of malignant tumors in head and neck. OSCC develops via stereotypical multistep processes. Abnormal TGF-β signaling has been proposed as one of the pathways leading to the carcinogenesis of OSCC [13]. Yet, it is largely unclear how abnormal TGF-β signaling contributes to carcinogenesis of OSCC.

We have previously reported various mutations of TβRII detected in 18 human OSCC samples [12]. In the present study, we investigated the I227T/N236D missense mutation located in the intracellular domain of TβRII, which harbors ACC (Thr) instead of ATC (Ile) at codon 227, and GAC (Asp) instead of AAC (Asn) at codon 236 (I227T/N236D). I227T/N236D mutation of TβRII, a novel mutation which has not been previously reported, was detected in the metastatic lymph node of one OSCC patient. We analyzed the effect of I227T/N236D TβRII mutation on TGF-β signaling and elucidated its role in the functional alterations leading to OSCC development.

## Methods

### Antibodies, plasmids, and other reagents

Matrigel (BD Matrigel Matrix, #356234) and type I collagen (#637–00653, Cellmatrix type I-A) were purchased from BD Biosciences (San Diego, CA, USA) and Nitta Gelatin (Osaka, Japan), respectively. Recombinant human TGF-β1 (#616455) was purchased from Calbiochem (Merck KGaA, Darmstadt, Germany). Curcumin (#C1386) and 3-(4,5-methylthiazol-2-yl)-2,5-diphenyltetrazolium bromide (MTT) (#M5655) were obtained from Sigma-Aldrich (Merck KGaA, Darmstadt, Germany). Antibodies against TβRII (#sc-220, 1:1000) were purchased from Santa Cruz Biotechnology (CA, USA). Antibodies against phospho-Smad2 (Ser465/467, #3101, 1:1000), Smad2 (#3122, 1:1000), cleaved Caspase-3 (#9661, 1:1000), cleaved PARP (#9541, 1:1000), epidermal growth factor receptor (EGFR) (#2232, 1:1000), phospho-EGFR (Tyr1068, #2234, 1:1000), phospho-Akt (Ser473, #9271, 1:1000), Akt (#9272, 1:1000) were purchased from Cell Signaling Technology, Inc. (Danvers, MA, USA). Antibodies against β-actin (#BS6007M, 1:5000) were obtained from Bioworld Technology, Inc. (St. Louis, MN, USA). Tyrphostin AG 1478 (#9842), a selective EGFR inhibitor, was purchased from Cell Signaling Technology, Inc. The cDNA for human TβRII (#RC519855) was purchased from Origene (Rockville, MD, USA). p3TP-lux (#11767) and pRL-TK (*Renilla reniformis* luciferase under thymidine kinase promoter) (#E2241) were obtained from Addgene (Cambridge, MA, USA) and Promega (Madison, WI, USA), respectively. Restriction enzymes were purchased from New England Biolabs (Beverly, MA, USA). Primers for cloning and mutagenesis were synthesized by Bioneer (Daejeon, South Korea). Phusion High-Fidelity DNA Polymerase (#F530S) for TβRII cloning and mutagenesis was supplied by Thermo Fisher Scientific, Inc. (Carlsbad, CA, USA).

### Cells and transient transfection

DR26 cells, mutant derivatives of Mv1Lu mink lung epithelial cells, which lack functional TβRII, were generously provided by Dr. J. Massague (Memorial Sloan-Kettering Cancer Center, New York, NY, USA). DR26 cells were cultured in Dulbecco’s modified Eagle’s medium (DMEM, #12-604F, Biowhittaker, Inc., Walkersville, MD, USA) supplemented with 10% fetal bovine serum (FBS, #26140079, Gibco, Thermo Fisher Scientific, Inc., Waltham, MA, USA) and 1% penicillin/streptomycin (#15140–163, Gibco, Thermo Fisher Scientific, Inc.) at 37 °C in the presence of 5% CO_2_. HSC-2 human OSCC cells were kindly provided by Prof. Takashi Muramatsu (Tokyo Dental College, Tokyo, Japan). HSC-2 cells were cultured in P medium (DMEM:Ham’s F-12; 3:1) supplemented with 10% FBS and 1% penicillin/streptomycin. Ham’s F-12 (#21700–075) was purchased from Thermo Fisher Scientific, Inc. Cells were transiently transfected using Lipofectamine 2000 (#11668–019, Invitrogen, Thermo Fisher Scientific, Inc.), following the manufacturer’s instructions.

### Mutagenesis

The I227T/N236D double mutant TβRII was obtained by sequential site-directed mutagenesis. First, a TβRII mutant with a threonine residue instead of isoleucine at amino acid 227 (I227T) was constructed by site-directed mutagenesis using PCR as described in our earlier report [10]. cDNA encoding full-length human TβRII was previously subcloned into pcDNA3 and pIRES2-EGFP vectors [10]. These plasmids were used as templates for PCR. Primers 5′-TTGGATCCGGGGTCTGCCATGGGTC-3′ (F-BamHI) and 5′-AATCTAGACTATTTGGTAGTGTTTAGGGAGC-3′ (R-XbaI) were used to clone TβRII in pcDNA3; 5′-TTCTCGAGGGGGTCTGCCATGGGTC-3′ (F-XhoI) and 5′-AAACCGCGGCTATTTGGTAGTGTTTAGGGAGCC-3′ (R-SacII) were used to clone TβRII in pIRES2-EGFP. Primers used for site-directed mutagenesis are as follows: 5′-GCCATCATCCTGGTAGATGACCGCTC-3′ (sense) and 5′-GAGCGGTCATCTACCAGGATGATGGC-3′ (antisense). The PCR was performed using primers, F-BamHI (F-XhoI) with antisense and sense with R-XbaI (R-SacII). Subsequently, using the products of first PCR, a second round of PCR was carried out using the primers, F-BamHI (F-XhoI) and R-XbaI (R-SacII). The mutant PCR product was ligated to the corresponding restriction enzyme sites of the vector to generate the I227T mutant TβRII in pcDNA3 or pIRES2-EGFP. The N236D mutant TβRII was constructed in a similar fashion, using primers, 5′-CAACATCAACCACATCACAGAGCTGCTG-3′ (sense) and 5′-CAGCAGCTCTGTGATGTGGTTGATGTTG-3′ (antisense). I227T mutant TβRII plasmids were used as templates for the second PCR mutagenesis. The integrity of the products was confirmed by sequencing.

### Construction of stable transfectant cells expressing TβRII

HSC-2 cells stably expressing wild-type or I227T/N236D mutant TβRII were constructed, as previously described [10]. After transfection with pIRES2-EGFP vector, wild-type TβRII, and I227T/N236D TβRII in pIRES2-EGFP, the cells were selected in a P medium containing 10% FBS and 400 ng/ml of G418 (#G8168, Sigma-Aldrich).

### Promoter-reporter assay

3TP-lux promoter-reporter was used to test the transcriptional activities induced by TβRII mutation. DR26 cells seeded in 24-well plates were transfected with 0.2 μ g of 3TP-lux promoter-reporter constructs, 0.2 μg of wild type or mutant TβRII in pcDNA3, and 0.5 μg of pRL-TK. At 24 h after the transfection, cells were stimulated with 1 ng/ml of TGF-β in DMEM containing 0.2% FBS. After incubation for 24 h, the firefly luciferase and *Renilla* luciferase activities were detected using Dual Luciferase Reporter Assay System (#E1910, Promega, Madison, WI, USA), according to the manufacturer’s instructions. Data were normalized to *Renilla* luciferase activity for evaluation of transfection efficiency.

### Cell viability assay

Cell viability was measured by MTT assay as previously described [10]. For cytotoxicity analysis, cells (1 × 10^4^) were seeded into the individual wells of a 96-well plate and treated with curcumin, AG1478 in the P medium containing 0.2% FBS. The optical density was measured at 570 nm using a microplate reader. All experiments were performed in triplicate.

### Western blot analysis

The cells (1 × 10^6^) were washed with cold PBS and lysed with Cell Lysis Buffer (#9803S, Cell Signaling Technology, Inc.) supplemented with phenylmethylsulfonyl fluoride (PMSF, # 78830, Cell Signaling Technology, Inc.). Protein concentration was determined using the Bicinchoninic Acid Protein Assay Kit (Pierce, Thermo Fisher Scientific, Inc.). Proteins (50 μg) were separated on 10% SDS-PAGE and transferred to PVDF membranes (#162–0177, Millipore, Billerica, MA, USA). The membranes were blocked in TBST (Tris-buffered saline with 0.5% of Triton X-100) containing 5% non-fat milk or BSA for 1 h at room temperature and incubated with appropriate primary antibodies at 4 °C overnight. After three washes with TBST, the membranes were incubated with horseradish peroxidase-conjugated anti-mouse (#7076S) or anti-rabbit (#7074S) secondary antibodies (Cell Signaling Technology, Inc.) for 1 h. Protein bands were visualized using chemiluminescence reagent (#W3651–024, GenDEPOT, Barker, TX, USA). The ImageJ software was used to quantify the signal intensity of protein bands.

### RNA interference

Two different small interfering RNA (siRNA) sequences were used to target EGFR. The siRNAs were synthesized by Bioneer and the sequences were as follows: siEGFR#1 sense, 5′-GCAAAGUGUGUAACGGAAUAGGUAU-3′ and antisense, 5′-AUACCUAUUCCGUUACACACUUUGC-3′; siEGFR#2 sense, 5′-GAGGAAAUAUGUACUACGA-3′ and antisense, 5′-UCGUAGUACAUAUUUCCUC-3′ (antisense). A negative control siRNA (#SN-1003) was also purchased from Bioneer. Cells (4 × 10^5^) were cultured in 60 mm dishes for 24 h. siRNA (100 nM) transfections were carried out using Lipofectamine RNAiMAX (#13778–075, Invitrogen, Thermo Fisher Scientific, Inc.), according to the manufacturers’ instructions. After 6 h of incubation, medium was replaced to fresh P medium containing 10% FBS and cells were cultured for an additional 42 h at 37 °C. For invasion assay, siRNA-transfected cells were stabilized for 18 h after the addition of fresh medium, and then, seeded into the upper chamber of transwell inserts.

### Transwell migration assay and scratch wound healing assay

Two methods, transwell migration assay and scratch wound healing assay, were used to evaluate cell motility. In transwell migration assay, cells (2 × 10^4^) were serum-starved for 16 h in P medium and seeded into the upper chamber of transwell inserts (8 μm pore size; #3422, Corning Costar, Lowell, MA, USA) with or without TGF-β1 (10 ng/ml). The lower chamber contained P medium supplemented with 10% FBS. After 24 h of incubation, cells that have migrated to the lower compartment were fixed in 10% formalin and stained with 0.025% crystal violet. The membrane filters were mounted on slides, and cells were counted under a microscope (five images per group; magnification, × 100). In scratch wound healing assay, cells (1 × 10^5^) were seeded into 6-well plates and transfected with siRNAs. At 48 h post-transfection, the cell monolayer was wounded with a 200 μl sterile pipette tip. Cells were washed once with PBS and fresh serum-free medium was added with or without TGF-β1 (10 ng/ml). Following 18 h of incubation, migrated cells were counted under a light microscope (magnification, × 100).

### Invasion assay

Transwell invasion assays were performed in a fashion similar to transwell migration assay except that the membrane inserts were precoated with 50 μl of 100 μg/ml Matrigel or 30 μl of 1.5 mg/ml Cellmatrix Type I-A. Briefly, after serum starvation for 16 h in P medium, cells (2 × 10^4^) were seeded into the transwell chamber inserts in the presence or absence of TGF-β1 (10 ng/ml). The P medium containing 1% FBS was added to the lower chamber. After 48 h of incubation, cells that have penetrated the filter were fixed and stained with 0.025% crystal violet, and counting under a light microscope (five images per group; magnification, × 100).

### Gelatin zymography

Gelatinase activities of matrix metalloproteinase-2 (MMP-2) and matrix metalloproteinase-9 (MMP-9) were analyzed by gelatin zymography as described previously [10]. Cells were seeded in 6-well plates and incubated in P medium containing 0.2% FBS in the presence of vehicle or TGF-β1 (10 ng/ml) for 24 h. Cell conditioned medium (15 μg protein) was subjected to 10% SDS-PAGE containing 0.1% gelatin under non-reducing conditions. After rinsing four times with 2.5% triton X-100 to remove SDS at room temperature, the gels were incubated overnight at 37 °C in a developing buffer (50 mM Tris-HCl, pH 7.5, 0.2 M NaCl, 5 mM CaCl_2_·2H_2_O, and 0.02% Brij-35, pH 7.6). The gels were stained with 0.5% Coomassie Brilliant Blue R-250 in 10% acetic acid and 50% methanol, and then destained with 50% methanol and 10% acetic acid solution.

### Annexin V/propidium iodide (PI) apoptosis assay

Cells (1 × 10^6^) were seeded in a 100 mm dish and incubated at 37 °C overnight. The cells were treated with 20 μM curcumin for 24 h, and then, stained with an FITC annexin V apoptosis detection kit (#556547, BD Pharmingen™, BD Biosciences, San Jose, CA, USA), according to the manufacturers’ instructions. Briefly, the cells were harvested and resuspended in binding buffer at a density of 1 × 10^6^ cells/ml. After the addition of annexin V-FITC (5 μl) and PI (20 μg/ml, 5 μl), the cells were incubated for 15 min at room temperature. Stained cells were analyzed by flow cytometry (BD FACSCanto II flow cytometer, BD Biosciences, Franklin Lakes, NJ, USA). Cytomics FC 500 with CXP software (Beckman Coulter, Fullerton, CA, USA) was used for data analysis.

### In vivo xenograft tumor growth assay

All the animal procedures were performed in accordance with a protocol approved by the Institutional Animal Care and Use Committee of Yonsei University. Five-week-old male BALV/c nude mice were purchased from Central Laboratory Animal Inc. (Seoul, Korea). All the mice were housed in the Yonsei Laboratory Animal Research Center and maintained in a pathogen-free environment (12 h light/dark cycle, 25 ± 2 °C; humidity, 50 ± 10%). Food and water were freely available. HSC-2 stable transfectant cells (2 × 10^5^ cells in 100 μl P medium) were orthotopically injected into the anterior tongue of the mice. Development of tongue tumors and weight loss was regularly monitored for 4 weeks. Tongue tissues containing tumor were fixed in 10% formalin neutral solution for at least 24 h at room temperature and embedded in paraffin. The tissues were sectioned into 4-μm-thick slices and stained with hematoxylin and eosin (H&E; #H-3502, Vector Laboratories, Inc., Burlingame, CA, USA). Tumor volume (TV) was calculated according to the formula: TV = (AxB^2^)/2, by measuring the diameters of the major axis (A) and the minor axis (B) of tumor [14].

### Immunohistochemistry

4-μm thick sections were cut from the paraffin blocks and immunostained as described [10]. Sections were deparaffinized in xylene, and hydrated in descending grades of ethanol. Sections were incubated with 3% hydrogen peroxide to inactivate endogenous peroxidase activities. After blocking in 1% normal goat serum (#S-1000, Vector Laboratories, Inc.) for 1 h, the sections were incubated with primary antibodies against phospho-EGFR (1:100) overnight at 4 °C, followed by incubation with biotinylated anti-rabbit IgG (#BA-1000, Vector Laboratories, Inc.) for 30 min. The sections were then incubated with horseradish peroxidase streptavidin (#SA-5004, Vector Laboratories, Inc.) for 30 min. The signal was developed using the 3,3′ diaminobenzidine tetrahydrochloride (DAB) kit (#SK-4100, Vector Laboratories, Inc.) and counterstained with Meyer’s hematoxylin (#H-3404, Vector Laboratories, Inc.). The staining intensity of the phosphorylated EGFR on the cell membrane was manually scored. An intensity scale ranging from 0 for no staining to 3+ for the most intense staining is used. H-Score is calculated using the following formula: H − Score = (%at 0) × 0 + (%at 1+) × 1 + (%at 2+) × 2 + (%at 3+) × 3.

### Statistical analysis

Mann-Whitney U test was performed for statistical analysis. All variables were analyzed from three independent experiments, and each experiment was performed at least in triplicate. The results are shown as the mean ± standard deviation. A *P*-value < 0.05 was considered as statistically significant.

## Results

### Analysis of canonical TGF-β signaling by I227T/N236D TβRII

**Fig. 1:**
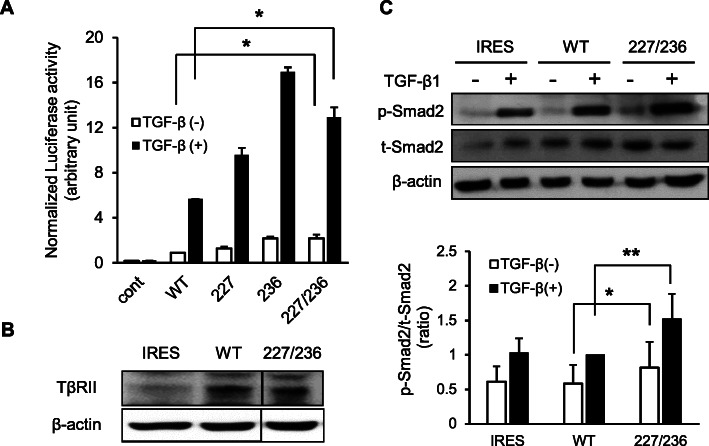
Effect of I227T/N236D TβRII mutation on TGF-β signaling. **a.** Transcriptional activities were assessed via luciferase assay after transfection with pcDNA3 vector (cont), wild-type (WT), I227T (227), N236D (236), and I227T/N236D (227/236) mutant TβRII constructs. DR26 cells were co-transfected with p3TP-lux promoter-reporter construct, pRL-TK, and TβRII constructs. Transfected cells were incubated for 24 h in DMEM supplemented with 0.2% FBS in the presence of vehicle (TGF-β (−)) or 1 ng/ml TGF-β1 (TGF-β (+)). Transfection efficiency was normalized using *Renilla* luciferase. Data represent mean ± standard deviation (*P < 0.05). **b.** Stable transfectant cells were constructed by transfection of pIRES2-EGFP vector (IRES), wild-type TβRII (WT), and I227T/N236D TβRII (227/236) constructs into HSC-2 cells. TβRII expression in stable cells was confirmed by western blotting. Protein samples of IRES, WT, and 227/236 were separated on the same gel and the protein bands were cropped. Uncropped blots were shown in Additional file 1: Figure S1. **c.** Stable HSC-2 cells harboring empty vector (IRES), wild-type TβRII (WT), and I227T/N236D TβRII (227/236) were mock-treated or treated with 10 ng/ml TGF-β1 for 18 h. Smad2 protein level (t-Smad2) and the phosphorylation level of Smad2 (p-Smad2) were determined by western blotting. Data represent mean ± standard deviation from three independent experiments (*P < 0.05, **P < 0.01). Full length immunoblots were shown in Additional file 2: Figure S2

We have previously identified various mutations of TβRII in human OSCC samples [12]. In this study, we analyzed the effect of one of these mutations, I227T/N236D, on tumor proliferation, migration, and invasion. Since I227T/N236D mutation was detected only in the metastatic lymph node, but not in the primary tumor, of an OSCC patient, we speculated this mutation could have been linked to functional alterations of TβRII leading to OSCC progression. First, we analyzed the transcriptional activities of wild-type and I227T/N236D mutant TβRII. 3TP-lux reporter contains three consecutive TPA response elements and TGF-β-responsive portion of human plasminogen activator inhibitor 1 (PAI-1) gene, linked to a luciferase reporter gene [5, 15]. We used 3TP-lux reporter to measure the transcriptional activities of wild-type and I227T/N236D mutant TβRII. DR26 cells without functional TβRII, were transiently co-transfected with 3TP-Lux, pRL-TK, and TβRII cDNAs, such as wild-type, I227T, N236D, and I227T/N236D TβRII. Cells were then treated with either vehicle or 1 ng/ml of TGF-β1 for 24 h. In the absence of TGF-β1, both wild-type and mutant TβRII expressions exerted basal level transcriptional activities. Upon TGF-β1 treatment, the transcriptional activity in all cells increased at least 5-fold when compared to mock treatment. When treated with TGF-β1, the transcriptional activity of mutant was significantly higher as compared to that of wild-type TβRII: 1.6-fold for I227T, 2.9-fold for N236D, and 2.1-fold for I227T/N236D mutant (*p* < 0.05) (Fig. 1a). These data suggest that I227T/N236D mutation of TβRII could enhance TGF-β signaling.

To determine the effect of I227T/N236D mutation on TGF-β signaling in human OSCC cells, HSC-2 cells were stably transfected with the empty vector, wild-type, and I227T/N236D mutant TβRII constructs. The expression of TβRII was higher in wild-type and mutant TβRII transfectant cells than in mock transfectant cells, indicating the expression of exogenous TβRII in these stable cells (Fig. 1b, Additional file 1: Figure S1). Next, we investigated whether exogenous TβRII expression activates TGF-β downstream signaling by analyzing Smad2 phosphorylation level (Fig. 1c, Additional file 2: Figure S2). Upon treatment with TGF-β, Smad2 phosphorylation level was significantly increased in mutant cells as compared to wild-type cells (*p* < 0.01). Taken together, these results strongly imply that the I227T/N236D mutation of TβRII enhances TGF-β signaling in human OSCC cells.

### Effects of I227T/N236D TβRII mutation in vivo

**Fig. 2:**
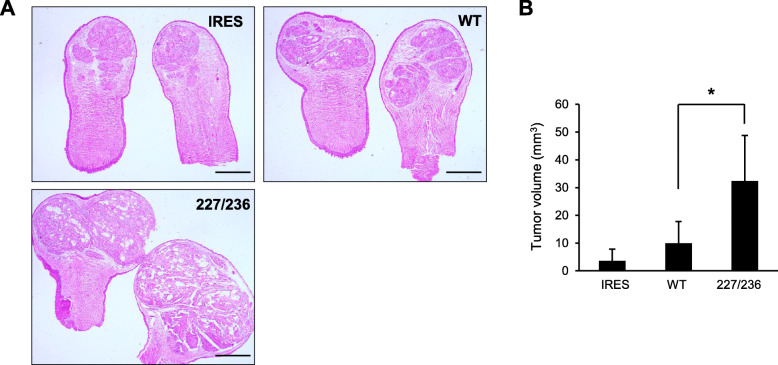
Effect of I227T/N236D TβRII mutation on xenograft tumor growth. **a.** Stable transfectant cells harboring an empty vector (IRES), wild-type TβRII (WT), or I227T/N236D TβRII (227/236) were orthotopically injected into the anterior tongue of mice. Tongue tumor sections were stained with H&E. Scale bar, 1 mm. **b.** Tongue tumor volume was measured at 4 weeks of injection. Data represent mean tumor volume ± standard deviation (* *P* < 0.05)

To elucidate the effect of upregulated TGF-β signaling by I227T/N236D mutation in HSC-2 cells, we measured the proliferative capacities of the stable cells in the presence or absence of TGF-β1. There was no significant difference in in vitro growth between wild type and I227T/N236D mutant cells regardless of the presence of TGF-β1 (Additional file 3: Figure S3). These findings indicate that HSC-2 cells are resistant to growth inhibition by TGF-β signaling, as previously reported [10]. Next, we used a human tumor xenograft mouse model to analyze the effect of I227T/N236D mutation on in vivo proliferative capabilities. Tongue tumors developed in all mice at 4 weeks post-injection into the anterior tongue (*n* = 5 to 8 mice per group). Mice injected with I227T/N236D mutant cells developed 7- and 3-fold larger tumors as compared to mice injected with IRES or wild type cells (*p* < 0.05), respectively (Fig. 2a and b). Taken together, these data indicate that enhanced TGF-β signaling by I227T/N236D mutation of TβRII promotes tumor growth in vivo.

### Migratory and invasive capabilities of I227T/N236D TβRII HSC-2 cells

**Fig. 3:**
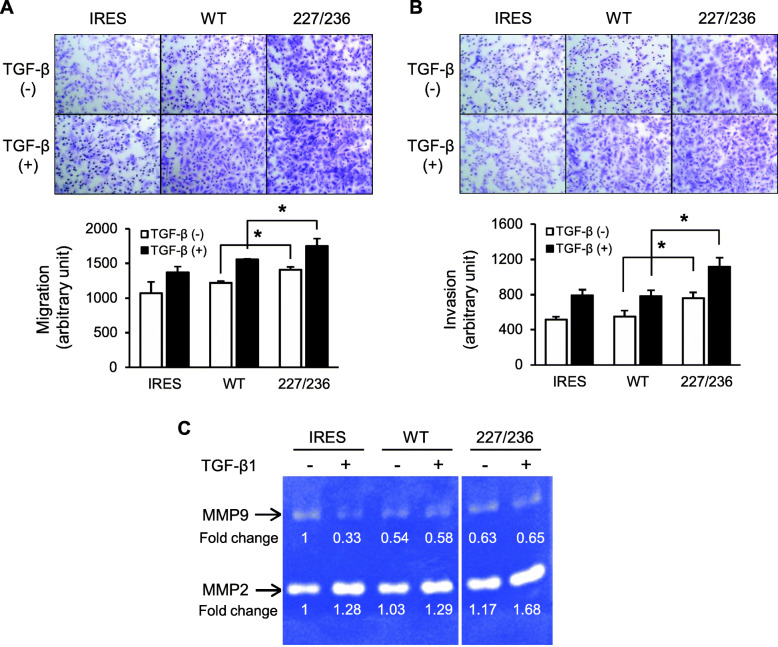
Migratory and invasive capabilities of stable HSC-2 cells expressing exogenous TβRII. **a** and **b.** Stable transfectant cells harboring an empty vector (IRES), wild-type TβRII (WT), or I227T/N236D TβRII (227/236) were seeded in a transwell chamber (**a**) or transwell chamber precoated with Matrigel (**b**). Cells that migrated to the other side of the chamber were counted after 24 h of incubation in the presence of vehicle (TGF-β1 (−)) or 10 ng/ml of TGF-β1 (TGF-β1 (+)); magnification, × 100. Data represent mean ± standard deviation (*P < 0.05). **c.** Gelatinolytic activities of MMP-2 and MMP-9 were assayed. Stable transfectant cells were incubated in P medium containing 0.2% FBS in the presence of vehicle (−) or 10 ng/ml of TGF-β1 (+) for 24 h. The conditioned medium was collected and subjected to gelatin zymography. Samples of IRES, WT, and 227/236 were assayed on the same gel and the images were cropped. IRES, empty vector; WT, wild-type TβRII; 227/236, I227T/N236D TβRII. Uncropped gelatin zymography was shown in Additional file 4: Figure S4

We next examined the invasive and migratory capabilities of I227T/N236D TβRII. First, a transwell migration assay was performed. As a chemo-attractant, the complete medium containing TGF-β1 (10 ng/ml) was used. In the absence of TGF-β1, migration of I227T/N236D cells was 1.2-fold higher than that of wild-type cells (*p* < 0.05). In the presence of TGF-β1, I227T/N236D cells still showed slightly higher migratory capabilities (1.1-fold) than wild-type TβRII cells (*p* < 0.05) (Fig. 3a). In filter-based invasion assay, I227T/N236D cells showed enhanced invasive capabilities as compared to wild-type TβRII cells (1.4-fold) regardless of the presence of TGF-β1 (*p* < 0.05) (Fig. 3b).

Transcriptional activation of MMP2 and MMP9 by TGF-β signaling is correlated with the migration and invasion of cancer cells [16]. MMP2 and MMP9 cleave the type IV and type V collagen and gelatin. MMP2 and MMP9 also convert latent TGF-β to active form, creating a positive feedback loop [17, 18]. Thus, we determined the activity of MMP2 and MMP9. MMP2 activity was elevated in I227T/N236D cells compared to wild-type cells by 1.3-fold in the presence of TGF-β1 (Fig. 3c, Additional file 4: Figure S4). However, MMP9 activity was decreased in wild-type cells and I227T/N236D cells, as compared to IRES. Taken together, these results indicate that I227T/N236D mutation of TβRII promotes cell migration and invasion, at least partly, by inducing MMP2 activity.

### I227T/N236D mutation confers resistance to apoptosis in HSC-2 cells

**Fig. 4:**
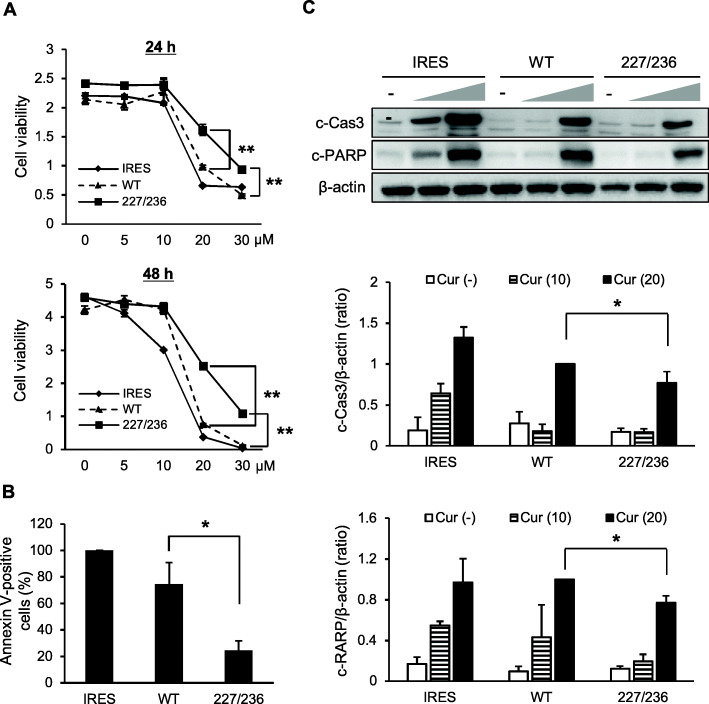
Curcumin-mediated apoptotic induction in cells stably expressing exogenous TβRII. **a.** Stable transfectant cells were cultured with vehicle or varying concentrations of curcumin (5–30 μM) for 24 h and 48 h, followed by MTT assay. Data represent mean ± standard deviation. (**P < 0.01) **b.** Stable transfectant cells harboring an empty vector (IRES), wild-type TβRII (WT), or I227T/N236D TβRII (227/236) were treated with 20 μM of curcumin for 24 h. Cells were stained with annexin V-FITC and PI, followed by flow cytometric analysis. Bars represent the population of annexin V-positive cells (mean ± standard deviation) (*P < 0.05). **c.** Cleaved caspase-3(c-Cas3) and cleaved PARP(c-PARP) were analyzed by western blotting. Stable transfectant cells were incubated in P medium containing 0.2% FBS in the presence of vehicle (−) or curcumin (10 and 20 μM) for 24 h. Data represent mean ± standard deviation from three independent experiments (*P < 0.05). IRES, empty vector; WT, wild-type TβRII; 227/236, I227T/N236D TβRII. Full length immunoblots were shown in Additional file 5: Figure S5

In an attempt to understand the differences between in vitro and in vivo proliferative capabilities of I227T/N236D TβRII mutant cells, cells were treated with curcumin to induce apoptosis since apoptosis is crucial for maintaining the balance between proliferation and death of normal cells under in vivo conditions. Curcumin possesses anti-proliferative and anti-carcinogenic properties and induces apoptosis of tumor cells [19–21]. Stable transfectant cells were treated with varying concentrations of curcumin. I227T/N236D mutant cells were more resistant to proliferation inhibition by curcumin than wild-type cells when treated for 24 or 48 h (*p* < 0.01) (Fig. 4a). To assess whether differential sensitivity to curcumin-mediated proliferation inhibition was related to apoptotic resistance, Annexin V staining was performed following treatment with curcumin. Consistent with the results of Fig. 4a, Annexin V-positive population was significantly smaller in I227T/N236D-mutant cells than in wild type cells after curcumin treatment (*p* < 0.05) (Fig. 4b). To confirm this result, the levels of cleaved caspase-3 and cleaved PARP were assayed by western blotting (Fig. 4c, Additional file 5: Figure S5). The levels of cleaved caspase-3 and cleaved PARP increased upon treatment with curcumin in a dose-dependent manner in all stable cells (*p* < 0.05). However, the levels of both cleaved caspase-3 and cleaved PARP were significantly lower in I227T/N236D mutant cells as compared to wild-type cells. Taken together, these results suggest that I227T/N236D mutation of TβRII confers apoptotic resistance to HSC-2 stable cells.

### EGFR activation by I227T/N236D mutation

**Fig. 5:**
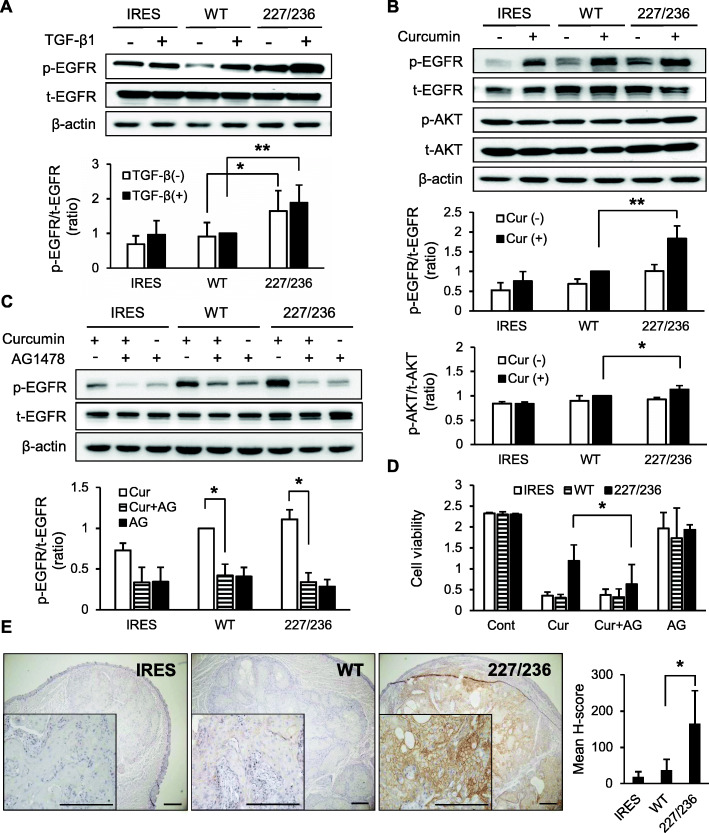
Curcumin-mediated EGFR activation in stable HSC-2 cells expressing exogenous TβRII. **a.** Stable HSC-2 cells were incubated in P medium containing 0.2% FBS in the presence of vehicle (−) or 10 ng/ml of TGF-β1 (+) for 24 h. EGFR protein level (t-EGFR) and phosphorylation of EGFR (p-EGFR) were detected by western blotting. Data represent mean ± standard deviation from three independent experiments (*P < 0.05, **P < 0.01). Full length immunoblots were shown in Additional file 6: Figure S6. **b.** Stable transfectant cells were incubated in P medium containing 0.2% FBS in the presence of vehicle (−) or 10 μ M curcumin (+) for 24 h. Activation of EGFR and AKT was detected by western blotting. t-EGFR, EGFR protein; p-EGFR, phosphorylated EGFR; t-AKT, AKT protein; p-AKT, phosphorylated AKT; Cur, curcumin. Data represent mean ± standard deviation from three independent experiments. (*P < 0.05, **P < 0.01). Full length immunoblots were shown in Additional file 7: Figure S7. **c** and **d**. Stable HSC-2 cells were incubated with 200 nM AG1478 (AG) for 30 min and treated with 10 μM curcumin (Cur) for 24 h. Activation of EGFR was determined by western blotting (**c**) Full length immunoblots were shown in Additional file 8: Figure S8. Cell viability was measured by MTT assay (**d**). Data represent mean ± standard deviation from three independent experiments (*P < 0.05). **e.** Immunohistochemical analysis of phosphorylated EGFR in xenograft tongue tumor tissues. Magnification, × 40 and × 200 (inset). Scale bar, 200 μm. Semiquantitative analysis of phosphorylated EGFR was performed using the H-score method. Data represent mean ± standard deviation (*P < 0.05). IRES, empty vector; WT, wild-type TβRII; 227/236, I227T/N236D TβRII

We next analyzed the activation of pro-survival molecules involved in TGF-β signaling cascade, such as EGFR and AKT. Upon treatment with TGF-β1 the phosphorylation of EGFR was enhanced in all transfectant cells (Fig. 5a, Additional file 6: Figure S6). Activation of EGFR was higher in I227T/N236D TβRII mutant cells as compared to IRES and wild-type cells in the presence of TGF-β1 (*p* < 0.01) indicating that enhanced TGF-β signaling results in EGFR activation in HSC-2 cells. Similar to TGF-β1 treatment, curcumin treatment also induced EGFR activation (*p* < 0.01), suggesting that curcumin induces not only apoptotic signaling but also pro-survival signaling. The I227T/N236D TβRII mutant cells showed higher activation of EGFR and AKT than wild-type cells when treated with curcumin (Fig. 5b, Additional file 7: Figure S7). These results indicate that I227T/N236D mutation of TβRII induces relatively stronger pro-survival signaling than the wild-type counterpart.

To confirm the involvement of enhanced EGFR activation in I227T/N236D mutant cell survival upon curcumin treatment, cells were treated with AG1478, an EGFR inhibitor. Curcumin-induced EGFR activation was efficiently blocked by AG1478 in all stable cells (*p* < 0.05) (Fig. 5c, Additional file 8: Figure S8). I227T/N236D mutant cell viability upon curcumin treatment was reduced to the level of IRES and wild-type cells following AG1478 treatment (*p* < 0.05), indicating that EGFR mediates the pro-survival signaling of I227T/N236D mutant cells (Fig. 5d). Taken together, these results suggest that the expression of I227T/N236D mutant TβRII confers resistance against curcumin-induced apoptosis in HSC-2 cells by enhancing pro-survival signaling of EGFR.

To verify the enhanced EGFR signaling by I227T/N236D mutation in vivo, we performed the immunohistochemical staining of phosphorylated EGFR in xenograft tongue tumor tissues. Phosphorylated EGFR was stained on the cell membrane of xenograft tumors (Fig. 5e). The staining intensity of the phosphorylated EGFR on the cell membrane was manually scored. Mean H-score of phosphorylated EGFR was significantly higher in I227T/N236D TβRII tongue tumor sections than in wild-type sections (*p* < 0.05). Taken together, our findings suggest that I227T/N236D TβRII mutation activates EGFR signaling, which could play a critical role in mutant cell survival.

### I227T/N236D TβRII mutation enhances migratory and invasive capacities via EGFR activation

**Fig. 6:**
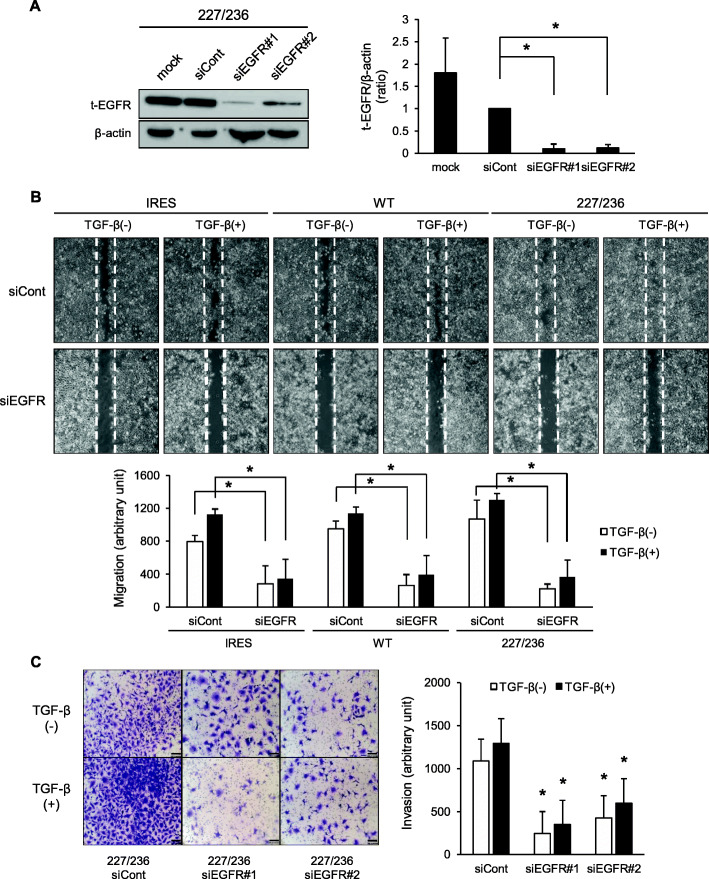
Effects of EGFR on migratory and invasive capabilities of I227T/N236D TβRII-expressing stable cells. **a.** I227T/N236D TβRII stable cells (227/236) were mock transfected (mock) or transfected with control siRNA (siCont) and two siRNAs targeting EGFR (siEGFR#1 and siEGFR#2). Protein level of EGFR was analyzed by western blotting. Data represent mean ± standard deviation from three independent experiments (*P < 0.05). Full length immunoblots were shown in Additional file 9: Figure S9. **b.** Stable cells were transfected with control siRNA (siCont) or co-transfected with two siRNAs targeting EGFR (siEGFR) and stabilized for 48 h, and then, a scratch wound was introduced in a cell monolayer, followed by incubation for 18 h in serum-free medium with vehicle (TGF- β (−)) or 10 ng/ml of TGF- β1 (TGF- β (+)). All migrated cells were counted. Data represent mean ± standard deviation (*P < 0.05, vs. control siRNA group). IRES, empty vector; WT, wild-type TβRII; 227/236, I227T/N236D TβRII. **c.** After siRNA transfection as in (**b**), I227T/N236D (227/236) cells were seeded in a transwell chamber precoated with Cellmatrix Type I-A. Cells that invaded the lower chamber were counted after 48 h of incubation in the presence of vehicle (TGF- β (−)) or 10 ng/ml of TGF- β1 (TGF- β (+)). Invaded cells were counted in five microscopic fields. Data represent mean ± standard deviation (*P < 0.05, vs. control siRNA group)

To analyze the causal relationship between EGFR activation and migratory and invasive capabilities of I227T/N236D mutant cells, the scratch wound healing and transwell cell invasion assays were performed after EGFR knockdown. EGFR was efficiently knockdowned by two different siRNAs targeting EGFR (*p* < 0.05) (Fig. 6a, Additional file 9: Figure S9). To determine the effect of EGFR on migratory capabilities induced by TβRII mutation, two siRNAs targeting EGFR were co-transfected into the stable cells. EGFR knockdown not only reduced the TGF-β signaling-promoted migratory capabilities but also significantly lowered the basal migratory capacities of all stable cells (*p* < 0.05) (Fig. 6b). This result suggests that EGFR signaling is crucial for migratory capacities of HSC-2 cells and also mediates TGF-β signaling-induced cell migration. The invasive capabilities of I227T/N236D cells were also significantly downregulated by EGFR knockdown (*p* < 0.05) (Fig. 6c). Taken together, these findings indicate that EGFR signaling plays a pivotal role in regulating migratory and invasive capacities of I227T/N236D TβRII mutant cells.

## Discussion

Various mutations of TβRII have been found in a variety of tumors [7, 11, 12, 22]. However, functional studies investigating specific mutations of TβRII from OSCC are rather scarce. The aim of the present study was to investigate the relationship between I227T/N236D mutation of TβRII and OSCC progression. The I227T/N236D mutation of TβRII resulted in enhanced TGF-β signaling, as revealed by higher transcriptional activities and Smad2 phosphorylation, as compared to wild-type. The transcriptional activity of TβRII was enhanced by I227T/N236D mutation regardless of the presence of TGF-β, suggesting that this mutation could elicit conformational changes to a higher affinity form for either TGF-β or TβRI. Another possibility for the higher transcriptional activity is that I227T/N236D mutation could modulate the rate of receptor internalization, leading to a prolonged and stronger TGF-β signaling as was previously observed under other TβRII mutations [10]. Underlying mechanism for the higher transcriptional activity by I227T/N236D mutation of TβRII remains to be clarified.

To analyze the effect of I227T/N236D mutation on TGF-β signaling in human OSCC cells, HSC-2 cells were used. We have previously shown that HSC-2 cells exhibited TGF-β responsiveness such as TGF-β-mediated Smad2/3 phosphorylation and EMT induction [10], suggesting that HSC-2 cells retain an intact TGF-β signaling machinery. Stable HSC-2 cells transfected with empty vector, wild-type TβRII, or I227T/N236D mutant TβRII did not exhibit significant differences in growth capabilities, which indicates that enhanced TGF-β signaling does not affect HSC-2 cell proliferation in vitro. However, in xenograft tumor growth assay, the I227T/N236D mutant TβRII exhibited around 3-fold higher tumor volume as compared to that of wild-type, implying that I227T/N236D mutation of TβRII promotes cell proliferation in vivo. To understand the discrepancy between in vivo and in vitro proliferative capacities, we focused on the differences in cell proliferation and death signaling among two-dimensional (2D) cultures, in vitro three-dimensional (3D) cultures, and in vivo growth conditions. It has previously been reported that cell proliferation rate is lower in 3D cell culture than in 2D culture as evidenced by the increased number of apoptotic cells and reduced S-phase cell population [23, 24]. Apoptosis also occurs during tumor progression in vivo, which plays a critical role by imposing a highly selective pressure enabling clonal expansion of aggressive sub-clones [25, 26]. The evasion of apoptosis in response to stress stimuli is thus an acquired hallmark of cancer. Differences in local availability of oxygen, nutrients, and signaling molecules within 3D culture or under in vivo conditions, are known factors that drive the variable cellular responses depending on their location [24]. We wondered, in this context, whether differential in vivo growth capacities of HSC-2 stable cells expressing wild-type and I227T/N236D mutant TβRII are due to varying susceptibilities to apoptotic stimuli of these cells. We thus analyzed apoptosis in 2D culture conditions after treatment with apoptotic inducers, such as curcumin. The I227T/N236D mutant stable cells were more resistant to apoptosis as compared to wild-type cells following treatment with apoptotic inducers. These results suggest that the enhanced TGF-β signaling of I227T/N236D mutant TβRII cells contribute to enhanced in vivo proliferative capacities of these cells via suppression of apoptosis.

In response to stress stimuli, cancer cells not only acquire resistance to apoptosis but also induce survival signaling that is tightly linked. The balance between survival and death signaling controls the tumor growth [26, 27]. Survival signaling involves abnormal activation of growth stimulating molecules, such as epidermal growth factor (EGF), platelet-derived growth factor (PDGF), nuclear factor κB (NF-κB), interleukins 2 and 3, mitogen-activated protein kinase (MAPK), and PI3K/AKT [25, 28, 29]. Our data showed that EGFR signaling was enhanced by curcumin in HSC-2 stable cells, and upregulation of TGF-β signaling further promoted curcumin-mediated EGFR signaling. These results indicate that EGFR signaling acts as an important survival mechanism in I227T/N236D TβRII stable cells. A number of reports have revealed that enhanced EGFR signaling disrupts the balance between cell growth and apoptosis during development of a variety of solid tumors [30, 31]. Abnormal activation of EGFR is known to occur mainly via overexpression, mutation, or autocrine stimulation of EGFR in cancers [30–32]. Transactivation of EGFR by TGF-β signaling has also been documented in breast, gastric cancer cells, and hepatocytes [33–36]. Taken together, our results indicate that EGFR activation via enhanced TGF-β signaling might play a critical role in tumor progression of OSCC by promoting cell survival.

Curcumin is a polyphenol found in the rhizome of *Curcuma longa* and exhibits nontoxic chemopreventive and chemotherapeutic activities [20]. Curcumin has been shown to induce apoptosis in a variety of cancer cells [37–40]. We used curcumin as an apoptotic inducer since these seemingly opposite effects could be gradually modulated by controlling the dose. Since curcumin has various molecular targets, diverse apoptotic and growth inhibitory signaling pathways are modulated by curcumin treatment. Several reports have shown that curcumin inhibits EGFR signaling via suppression of EGFR expression, induction of EGFR degradation, inhibition of kinase activity of EGFR, and modulation of EGFR dimerization in lung adenocarcinoma, colon cancer, and epidermoid carcinoma cell lines [19, 21, 41]. Curcumin induced apoptosis of HSC-2 cells at a concentration of 20 μM in 24 h. However, curcumin concentrations lower than 20 μM did not induce significant apoptosis in 24 h, but activated EGFR in all stable cells. We speculated that the exposure of HSC-2 cells to low concentrations of curcumin can activate pro-survival EGFR signaling as well as death signaling. EGFR signaling was higher in HSC-2 cells expressing I227T/N236D mutant TβRII than in wild-type counterpart. Concomitant with higher EGFR activation, the level of cleaved caspase-3 was lower in mutant cells than in wild-type cells upon curcumin treatment. AG1478, a pharmacological inhibitor of EGFR, efficiently suppressed EGFR activation, which led to the abrogation of apoptotic resistance of I227T/N236D mutant cells. Taken together, these results suggest that EGFR activation via enhanced TGF-β signaling is critical for the evasion of HSC-2 stable cells from apoptosis, which further supports the notion that I227T/N236D mutation of TβRII plays an important role in cell proliferation in vivo.

TGF-β treatment enhanced the migratory and invasive activities of HSC-2 cells. The I227T/N236D mutant TβRII cells exhibited higher migratory and invasive capabilities compared to wild-type cells regardless of the presence of TGF-β. To validate the role of EGFR in regulating migratory and invasive capabilities of I227T/N236D TβRII stable cells, EGFR was knockdowned in mutant cells. EGFR silencing dramatically reduced migratory and invasive capabilities of I227T/N236D TβRII cells, indicating that EGFR signaling plays a pivotal role in cell migration and invasion as well as in proliferation of OSCC.

In summary, our findings suggest that enhanced TGF-β signaling via I227T/N236D mutation of TβRII promotes EGFR activation, leading to tumorigenesis of OSCC by enhancing the hallmark features of cancer, such as apoptotic resistance and more invasive phenotypic changes.

## Conclusion

This study shows the relationship between a novel TβRII mutation detected in the metastatic lymph node of a patient, I227T/N236D, on oral squamous cell carcinoma progression. We identified that I227T/N236D mutation can enhance proliferative capacities and apoptotic resistance via upregulated TGF-β signaling. Furthermore, EGFR activation is involved in proliferative capabilities and apoptotic resistance as well as in migratory and invasive capacities of I227T/N236D TβRII cells. These findings will provide the development of new therapeutic approach for metastatic OSCC patients.

## Supplementary Information

**Additional file 1: Figure S1.** Full length immunoblots of TβRII and β-actin in Fig. 1b. Stable transfectant cells were constructed by transfection of pIRES2-EGFP vector (IRES), wild-type TβRII (WT), and I227T/N236D TβRII (227/236) constructs into HSC-2 cells. TβRII expression in stable cells was confirmed by western blotting. Protein samples were run in two identical sets and transferred to PVDF membranes. Protein samples of IRES, WT, and 227/236 were separated on the same gel and the corresponding protein bands were cropped. The red rectangle represents the cropping area.

**Additional file 2: Figure S2.** Full length immunoblots of p-Samd2, t-Smad2 and β-actin in Fig. 1c. Stable HSC-2 cells harboring empty vector (IRES), wild-type TβRII (WT), and I227T/N236D TβRII (227/236) were mock-treated or treated with 10 ng/ml TGF-β1 for 18 h. Smad2 protein level (t-Smad2) and the phosphorylation level of Smad2 (p-Smad2) were determined by western blotting. Protein samples were run in three identical sets and transferred to PVDF membranes. Membranes were probed with p-Smad2 antibodies, Smad2 antibodies and β-actin antibodies, respectively.

**Additional file 3: Figure S3.** The growth of stable transfectant cells harboring an empty vector (IRES), wild-type TβRII (WT), or I227T/N236D TβRII (227–236). The stable cells were cultured in the presence of vehicle (−) or 10 ng/mL of TGF-β1 (+) for up to 4 days. Cell proliferation was measured by MTT assay. Data represent mean ± standard deviation. *P < 0.05 (mutant vs. wild-type).

**Additional file 4: Figure S4.** Full length gelatin zymography in Fig. 3c. Gelatinolytic activities of MMP-2 and MMP-9 were assayed. Stable transfectant cells were incubated in P medium containing 0.2% FBS in the presence of vehicle (−) or 10 ng/ml of TGF-β1 (+) for 24 h. The conditioned medium was collected and subjected to gelatin zymography. Samples of IRES, WT, and 227/236 were assayed on the same gel and the corresponding images were cropped. The red rectangle represents the cropping area.

**Additional file 5: Figure S5.** Full length immunoblots of Cleaved caspase-3(c-Cas3) and cleaved PARP(c-PARP) and β-actin in Fig. 4c. Stable transfectant cells were incubated in P medium containing 0.2% FBS in the presence of vehicle (−) or curcumin (10 μM and 20 μM) for 24 h. Protein samples were run in three identical sets and transferred to PVDF membranes. Membranes were probed with c-Cas3 antibodies, c-PARP antibodies and β-actin antibodies, respectively.

**Additional file 6: Figure S6.** Full length immunoblots of EGFR protein level (t-EGFR), phosphorylation of EGFR (p-EGFR) and β-actin in Fig. 5a. Stable HSC-2 cells were incubated in P medium containing 0.2% FBS in the presence of vehicle (−) or 10 ng/ml of TGF-β1 (+) for 24 h. EGFR protein level (t-EGFR) and phosphorylation of EGFR (p-EGFR) were detected by western blotting. Same protein samples were run three times and transferred to PVDF membranes. Each membrane was cut into two parts and the membrane parts harboring target protein bands were probes with the corresponding antibodies.

**Additional file 7: Figure S7.** Full length immunoblots of EGFR protein level (t-EGFR), phosphorylation of EGFR (p-EGFR), AKT protein level (t-AKT), phosphorylation of AKT (p-AKT) and β-actin in Fig. 5b. Stable transfectant cells were incubated in P medium containing 0.2% FBS in the presence of vehicle (−) or 10 μ M curcumin (+) for 24 h. Activation of EGFR and AKT was detected by western blotting. Protein samples were run in three identical sets and transferred to PVDF membranes. First membrane was probed with p-EGFR antibodies. Second membrane was probed with p-AKT, followed by stripping and reprobing with AKT antibodies. Third membrane was cut into two parts. Upper part was probed with EGFR antibodies and the lower part was probed with β-actin antibodies.

**Additional file 8: Figure S8.** Full length immunoblots of EGFR protein level (t-EGFR), phosphorylation of EGFR (p-EGFR) and β-actin in Fig. 5c. Stable HSC-2 cells were incubated with 200 nM AG1478 (AG) for 30 min and treated with 10 μM curcumin (Cur) for 24 h. Activation of EGFR was determined by western blotting. Protein samples were run in two identical sets and transferred to PVDF membranes. First membrane was probed with p-EGFR antibodies. Second membrane was cut into two parts. Upper part was probed with EGFR antibodies and the lower part was probed with β-actin antibodies.

**Additional file 9: Figure S9.** Full length immunoblots of EGFR protein level (t-EGFR) and β-actin in Fig. 6a. I227T/N236D TβRII stable cells (227/236) were mock transfected (mock) or transfected with control siRNA (siCont) and two siRNAs targeting EGFR (siEGFR#1 and siEGFR#2). Protein level of EGFR was analyzed by western blotting. Protein samples were run in two identical sets and transferred to PVDF membranes. First membrane was probed with EGFR antibodies and the second membrane was probed with β-actin antibodies.

## Data Availability

The datasets generated in and/or analyzed from the current study are available in Genbank, MW262807.

## Abbreviations

TGF-β: Transforming growth factor-ß
TβRII: Transforming growth factor-ß receptor II
OSCC: Oral squamous cell carcinoma
EGFR: Epidermal growth factor receptor
MAPKs: Mitogen-activated protein kinases
EMT: Epithelial-mesenchymal transition
MMP: Matrix metalloproteinase
PDGF: Platelet-derived growth factor
NF-κB: Nuclear factor κB
