# Supplementary material for: A novel TGF-β receptor II mutation (I227T/N236D) promotes aggressive phenotype of oral squamous cell carcinoma via enhanced EGFR signaling
Source: BMC Cancer. 2020 Nov 27;20:1163. doi: 10.1186/s12885-020-07669-5 (PMC7694911; doi:10.1186/s12885-020-07669-5)
Supplement: Supplementary file 1 — Additional file 1: Figure S1. Full length immunoblots of TβRII and β-actin in Fig. 1b. Stable transfectant cells were constructed by transfection of pIRES2-EGFP vector (IRES), wild-type TβRII (WT), and I227T/N236D TβRII (227/236) constructs into HSC-2 cells. TβRII expression in stable cells was confirmed by western blotting. Protein samples were run in two identical sets and transferred to PVDF membranes. Protein samples of IRES, WT, and 227/236 were separated on the same gel and the corresponding protein bands were cropped. The red rectangle represents the cropping area. [file 12885_2020_7669_MOESM1_ESM.pdf]

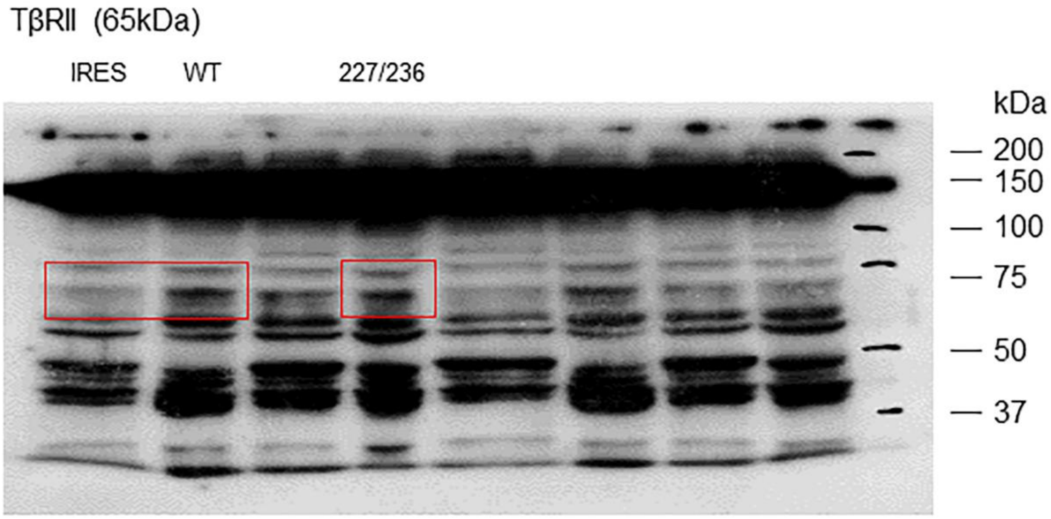

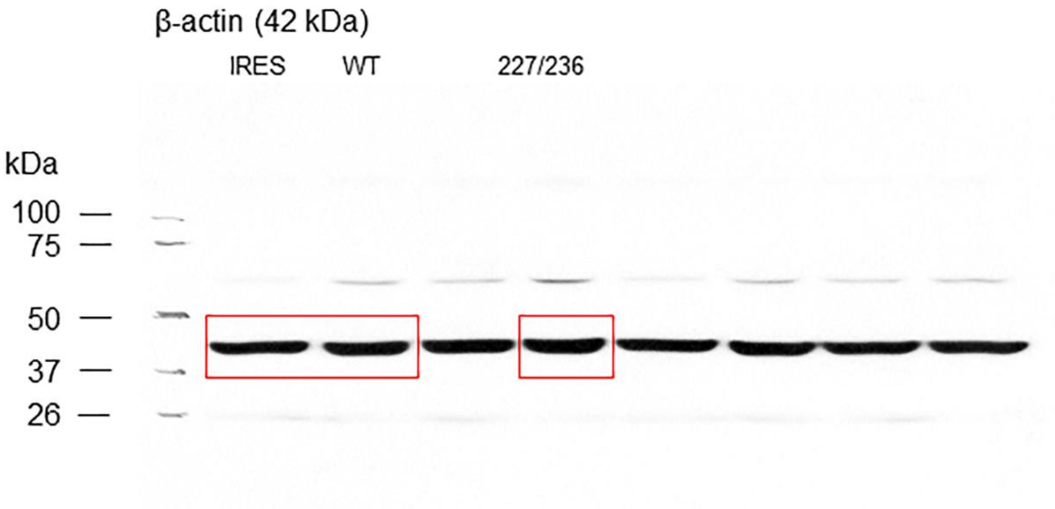

Fig. S1. Full length immunoblots of TβRII and β-actin in **Fig. 1b**. Stable transfectant cells were constructed by transfection of pIRES2-EGFP vector (IRES), wild-type TβRII (WT), and I227T/N236D TβRII (227/236) constructs into HSC-2 cells. TβRII expression in stable cells was confirmed by western blotting. Protein samples were run in two identical sets and transferred to PVDF membranes. Protein samples of IRES, WT, and 227/236 were separated on the same gel and the corresponding protein bands were cropped. The red rectangle represents the cropping area.
