# Supplementary material for: A novel TGF-β receptor II mutation (I227T/N236D) promotes aggressive phenotype of oral squamous cell carcinoma via enhanced EGFR signaling
Source: BMC Cancer. 2020 Nov 27;20:1163. doi: 10.1186/s12885-020-07669-5 (PMC7694911; doi:10.1186/s12885-020-07669-5)
Supplement: Supplementary file 2 — Additional file 2: Figure S2. Full length immunoblots of p-Samd2, t-Smad2 and β-actin in Fig. 1c. Stable HSC-2 cells harboring empty vector (IRES), wild-type TβRII (WT), and I227T/N236D TβRII (227/236) were mock-treated or treated with 10 ng/ml TGF-β1 for 18 h. Smad2 protein level (t-Smad2) and the phosphorylation level of Smad2 (p-Smad2) were determined by western blotting. Protein samples were run in three identical sets and transferred to PVDF membranes. Membranes were probed with p-Smad2 antibodies, Smad2 antibodies and β-actin antibodies, respectively. [file 12885_2020_7669_MOESM2_ESM.pdf]

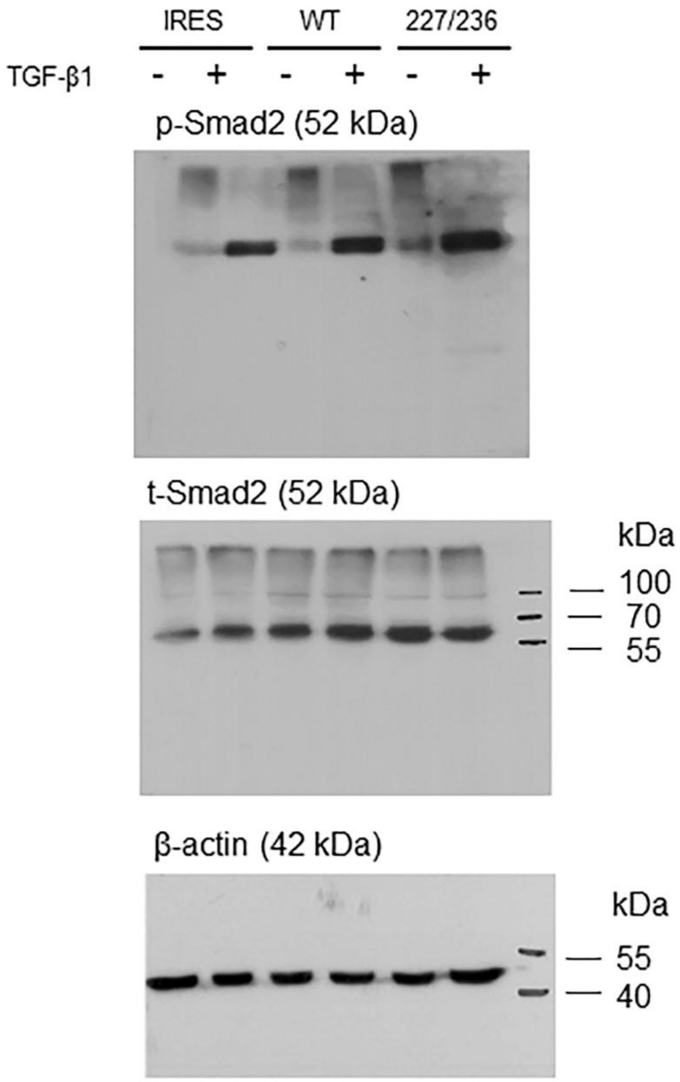


**Fig. S2.** Full length immunoblots of p-Samd2, t-Smad2 and β-actin in **Fig. 1c**. Stable HSC-2 cells harboring empty vector (IRES), wild-type TβRII (WT), and I227T/N236D TβRII (227/236) were mock-treated or treated with 10 ng/ml TGF-β1 for 18 h. Smad2 protein level (t-Smad2) and the phosphorylation level of Smad2 (p-Smad2) were determined by western blotting. Protein samples were run in three identical sets and transferred to PVDF membranes. Membranes were probed with p-Smad2 antibodies, Smad2 antibodies and β-actin antibodies, respectively.
