# Supplementary material for: A novel TGF-β receptor II mutation (I227T/N236D) promotes aggressive phenotype of oral squamous cell carcinoma via enhanced EGFR signaling
Source: BMC Cancer. 2020 Nov 27;20:1163. doi: 10.1186/s12885-020-07669-5 (PMC7694911; doi:10.1186/s12885-020-07669-5)
Supplement: Supplementary file 3 — Additional file 3: Figure S3. The growth of stable transfectant cells harboring an empty vector (IRES), wild-type TβRII (WT), or I227T/N236D TβRII (227–236). The stable cells were cultured in the presence of vehicle (−) or 10 ng/mL of TGF-β1 (+) for up to 4 days. Cell proliferation was measured by MTT assay. Data represent mean ± standard deviation. *P < 0.05 (mutant vs. wild-type). [file 12885_2020_7669_MOESM3_ESM.pdf]

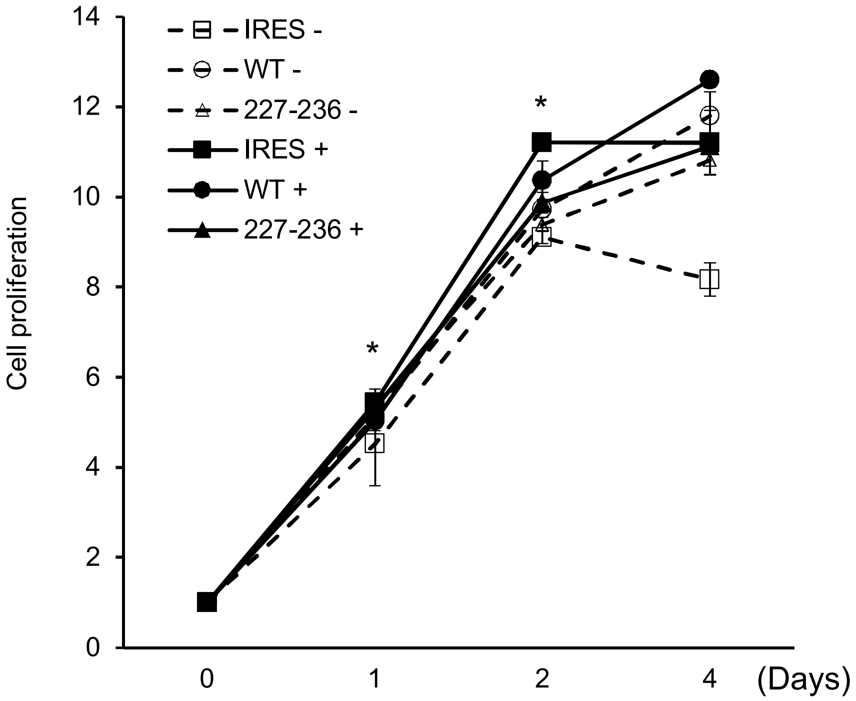

Fig. S3. The growth of stable transfectant cells harboring an empty vector (IRES), wild-type TβRII (WT), or I227T/N236D TβRII (227-236). The stable cells were cultured in the presence of vehicle (-) or 10 ng/mL of TGF-β1 (+) for up to 4 days. Cell proliferation was measured by MTT assay. Data represent mean ± standard deviation. *$P < 0.05$ (mutant vs. wild-type).
