# Supplementary material for: A novel TGF-β receptor II mutation (I227T/N236D) promotes aggressive phenotype of oral squamous cell carcinoma via enhanced EGFR signaling
Source: BMC Cancer. 2020 Nov 27;20:1163. doi: 10.1186/s12885-020-07669-5 (PMC7694911; doi:10.1186/s12885-020-07669-5)
Supplement: Supplementary file 4 — Additional file 4: Figure S4. Full length gelatin zymography in Fig. 3c. Gelatinolytic activities of MMP-2 and MMP-9 were assayed. Stable transfectant cells were incubated in P medium containing 0.2% FBS in the presence of vehicle (−) or 10 ng/ml of TGF-β1 (+) for 24 h. The conditioned medium was collected and subjected to gelatin zymography. Samples of IRES, WT, and 227/236 were assayed on the same gel and the corresponding images were cropped. The red rectangle represents the cropping area. [file 12885_2020_7669_MOESM4_ESM.pdf]

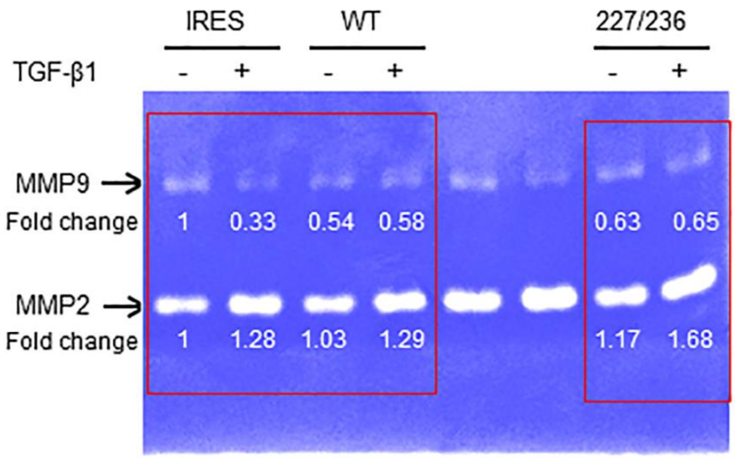

Fig. S4. Full length gelatin zymography in **Fig. 3c**. Gelatinolytic activities of MMP-2 and MMP-9 were assayed. Stable transfectant cells were incubated in P medium containing 0.2% FBS in the presence of vehicle (-) or 10 ng/ml of TGF-β1 (+) for 24 h. The conditioned medium was collected and subjected to gelatin zymography. Samples of IRES, WT, and 227/236 were assayed on the same gel and the corresponding images were cropped. The red rectangle represents the cropping area.
