# Supplementary material for: A novel TGF-β receptor II mutation (I227T/N236D) promotes aggressive phenotype of oral squamous cell carcinoma via enhanced EGFR signaling
Source: BMC Cancer. 2020 Nov 27;20:1163. doi: 10.1186/s12885-020-07669-5 (PMC7694911; doi:10.1186/s12885-020-07669-5)
Supplement: Supplementary file 5 — Additional file 5: Figure S5. Full length immunoblots of Cleaved caspase-3(c-Cas3) and cleaved PARP(c-PARP) and β-actin in Fig. 4c. Stable transfectant cells were incubated in P medium containing 0.2% FBS in the presence of vehicle (−) or curcumin (10 μM and 20 μM) for 24 h. Protein samples were run in three identical sets and transferred to PVDF membranes. Membranes were probed with c-Cas3 antibodies, c-PARP antibodies and β-actin antibodies, respectively. [file 12885_2020_7669_MOESM5_ESM.pdf]

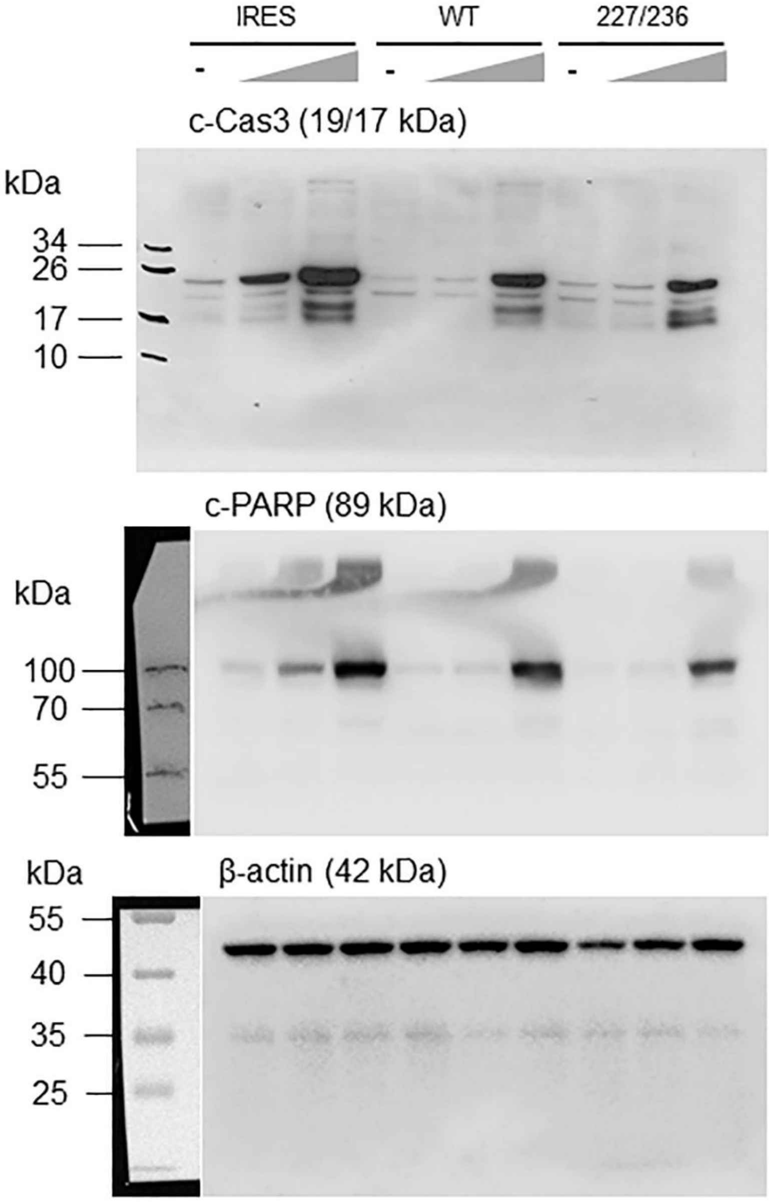

Fig. S5. Full length immunoblots of Cleaved caspase-3(c-Cas3) and cleaved PARP(c-PARP) and β-actin in **Fig. 4c**. Stable transfectant cells were incubated in P medium containing 0.2% FBS in the presence of vehicle (-) or curcumin (10 μM and 20 μM) for 24 h. Protein samples were run in three identical sets and transferred to PVDF membranes. Membranes were probed with c-Cas3 antibodies, c-PARP antibodies and β-actin antibodies, respectively.
