# Supplementary material for: A novel TGF-β receptor II mutation (I227T/N236D) promotes aggressive phenotype of oral squamous cell carcinoma via enhanced EGFR signaling
Source: BMC Cancer. 2020 Nov 27;20:1163. doi: 10.1186/s12885-020-07669-5 (PMC7694911; doi:10.1186/s12885-020-07669-5)
Supplement: Supplementary file 7 — Additional file 7: Figure S7. Full length immunoblots of EGFR protein level (t-EGFR), phosphorylation of EGFR (p-EGFR), AKT protein level (t-AKT), phosphorylation of AKT (p-AKT) and β-actin in Fig. 5b. Stable transfectant cells were incubated in P medium containing 0.2% FBS in the presence of vehicle (−) or 10 μ M curcumin (+) for 24 h. Activation of EGFR and AKT was detected by western blotting. Protein samples were run in three identical sets and transferred to PVDF membranes. First membrane was probed with p-EGFR antibodies. Second membrane was probed with p-AKT, followed by stripping and reprobing with AKT antibodies. Third membrane was cut into two parts. Upper part was probed with EGFR antibodies and the lower part was probed with β-actin antibodies. [file 12885_2020_7669_MOESM7_ESM.pdf]

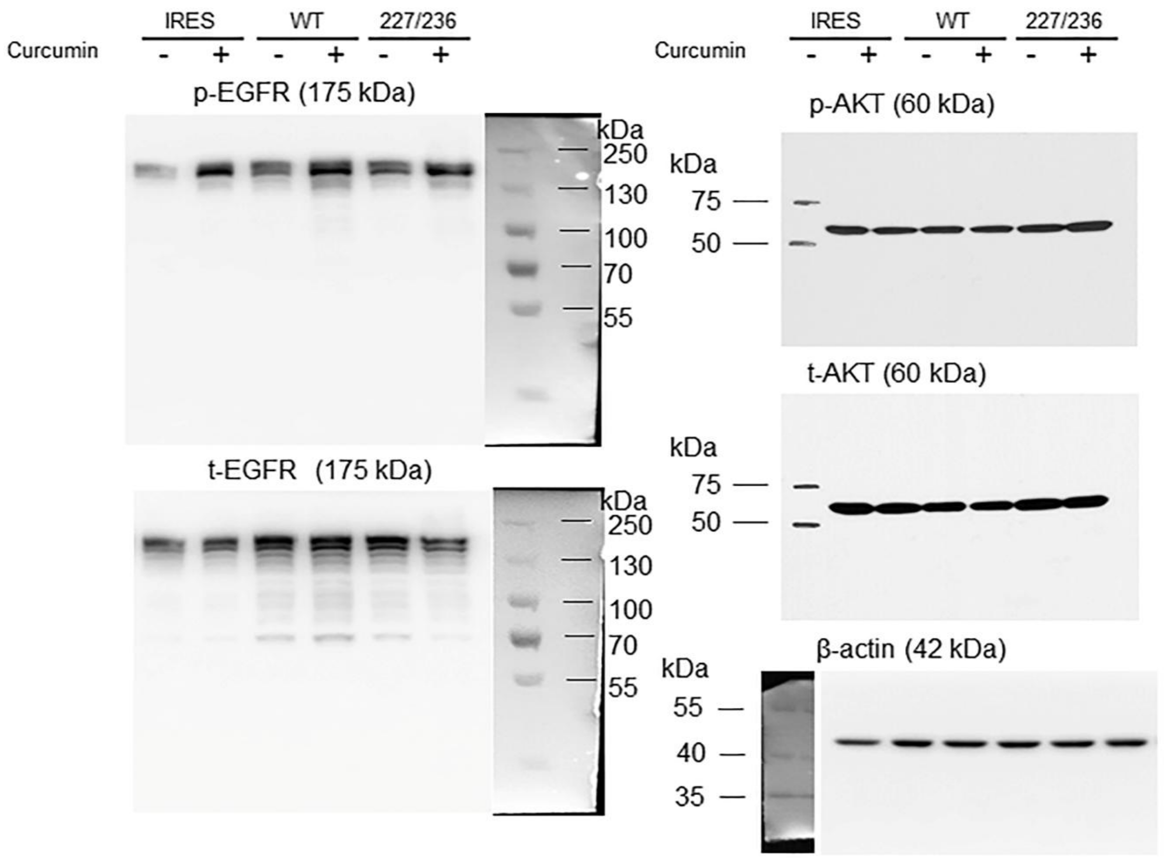

Fig. S7. Full length immunoblots of EGFR protein level (t-EGFR), phosphorylation of EGFR (p-EGFR), AKT protein level (t-AKT), phosphorylation of AKT (p-AKT) and β-actin in **Fig. 5b**. Stable transfectant cells were incubated in P medium containing 0.2% FBS in the presence of vehicle (-) or 10 μ M curcumin (+) for 24 h. Activation of EGFR and AKT was detected by western blotting. Protein samples were run in three identical sets and transferred to PVDF membranes. First membrane was probed with p-EGFR antibodies. Second membrane was probed with p-AKT, followed by stripping and reprobing with AKT antibodies. Third membrane was cut into two parts. Upper part was probed with EGFR antibodies and the lower part was probed with β-actin antibodies.
