# Supplementary material for: A novel TGF-β receptor II mutation (I227T/N236D) promotes aggressive phenotype of oral squamous cell carcinoma via enhanced EGFR signaling
Source: BMC Cancer. 2020 Nov 27;20:1163. doi: 10.1186/s12885-020-07669-5 (PMC7694911; doi:10.1186/s12885-020-07669-5)
Supplement: Supplementary file 8 — Additional file 8: Figure S8. Full length immunoblots of EGFR protein level (t-EGFR), phosphorylation of EGFR (p-EGFR) and β-actin in Fig. 5c. Stable HSC-2 cells were incubated with 200 nM AG1478 (AG) for 30 min and treated with 10 μM curcumin (Cur) for 24 h. Activation of EGFR was determined by western blotting. Protein samples were run in two identical sets and transferred to PVDF membranes. First membrane was probed with p-EGFR antibodies. Second membrane was cut into two parts. Upper part was probed with EGFR antibodies and the lower part was probed with β-actin antibodies. [file 12885_2020_7669_MOESM8_ESM.pdf]

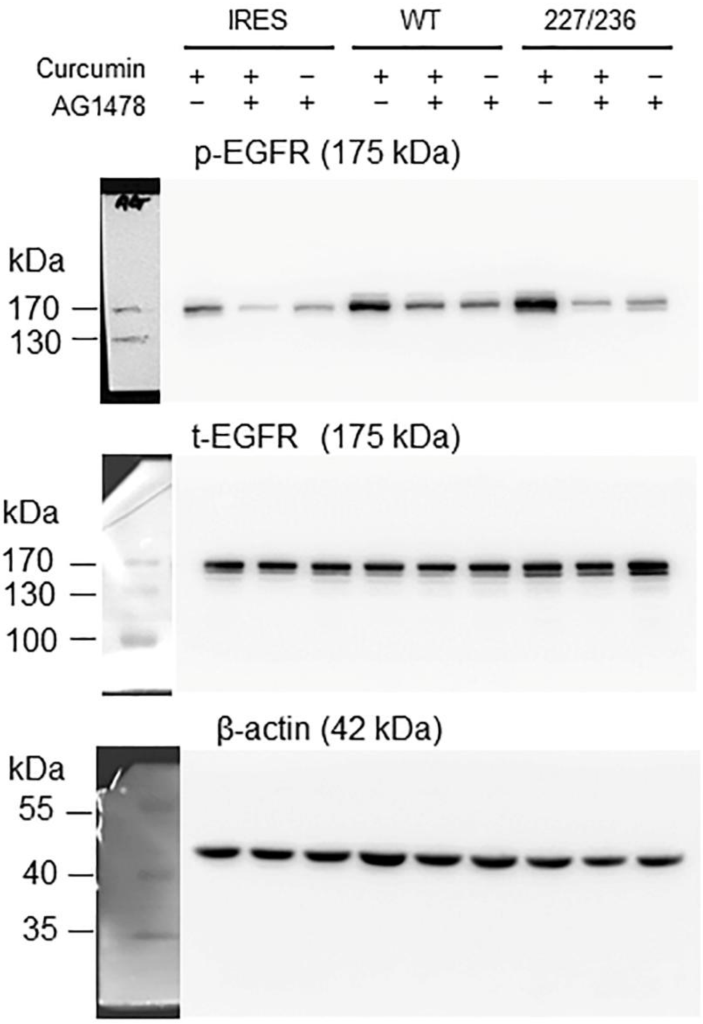

Fig. S8. Full length immunoblots of EGFR protein level (t-EGFR), phosphorylation of EGFR (p-EGFR) and β-actin in **Fig. 5c**. Stable HSC-2 cells were incubated with 200 nM AG1478 (AG) for 30 min and treated with 10 µM curcumin (Cur) for 24 h. Activation of EGFR was determined by western blotting. Protein samples were run in two identical sets and transferred to PVDF membranes. First membrane was probed with p-EGFR antibodies. Second membrane was cut into two parts. Upper part was probed with EGFR antibodies and the lower part was probed with β-actin antibodies.
