# Supplementary material for: A novel TGF-β receptor II mutation (I227T/N236D) promotes aggressive phenotype of oral squamous cell carcinoma via enhanced EGFR signaling
Source: BMC Cancer. 2020 Nov 27;20:1163. doi: 10.1186/s12885-020-07669-5 (PMC7694911; doi:10.1186/s12885-020-07669-5)
Supplement: Supplementary file 9 — Additional file 9: Figure S9. Full length immunoblots of EGFR protein level (t-EGFR) and β-actin in Fig. 6a. I227T/N236D TβRII stable cells (227/236) were mock transfected (mock) or transfected with control siRNA (siCont) and two siRNAs targeting EGFR (siEGFR#1 and siEGFR#2). Protein level of EGFR was analyzed by western blotting. Protein samples were run in two identical sets and transferred to PVDF membranes. First membrane was probed with EGFR antibodies and the second membrane was probed with β-actin antibodies. [file 12885_2020_7669_MOESM9_ESM.pdf]

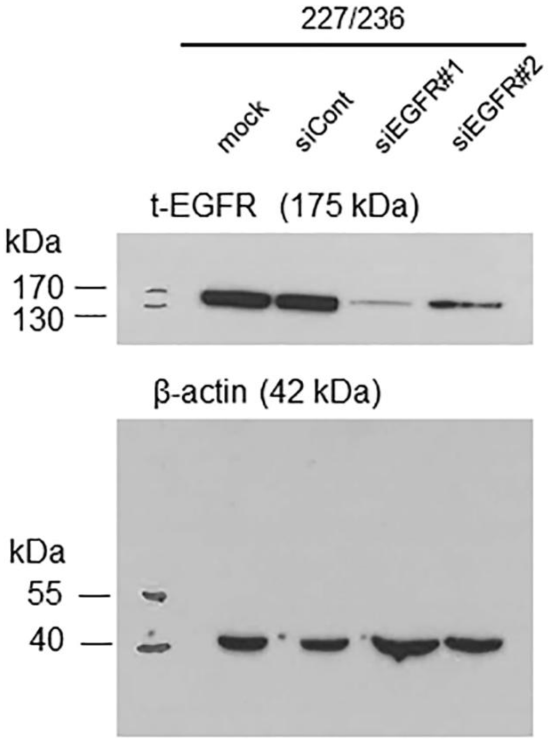

Fig. S9. Full length immunoblots of EGFR protein level (t-EGFR) and β-actin in **Fig. 6a**. I227T/N236D TβRII stable cells (227/236) were mock transfected (mock) or transfected with control siRNA (siCont) and two siRNAs targeting EGFR (siEGFR#1 and siEGFR#2). Protein level of EGFR was analyzed by western blotting. Protein samples were run in two identical sets and transferred to PVDF membranes. First membrane was probed with EGFR antibodies and the second membrane was probed with β-actin antibodies.
