# Supplementary material for: A novel TGF-β receptor II mutation (I227T/N236D) promotes aggressive phenotype of oral squamous cell carcinoma via enhanced EGFR signaling
Source: BMC Cancer. 2020 Nov 27;20:1163. doi: 10.1186/s12885-020-07669-5 (PMC7694911; doi:10.1186/s12885-020-07669-5)
Supplement: Supplementary file 6 — Additional file 6: Figure S6. Full length immunoblots of EGFR protein level (t-EGFR), phosphorylation of EGFR (p-EGFR) and β-actin in Fig. 5a. Stable HSC-2 cells were incubated in P medium containing 0.2% FBS in the presence of vehicle (−) or 10 ng/ml of TGF-β1 (+) for 24 h. EGFR protein level (t-EGFR) and phosphorylation of EGFR (p-EGFR) were detected by western blotting. Same protein samples were run three times and transferred to PVDF membranes. Each membrane was cut into two parts and the membrane parts harboring target protein bands were probes with the corresponding antibodies. [file 12885_2020_7669_MOESM6_ESM.pdf]

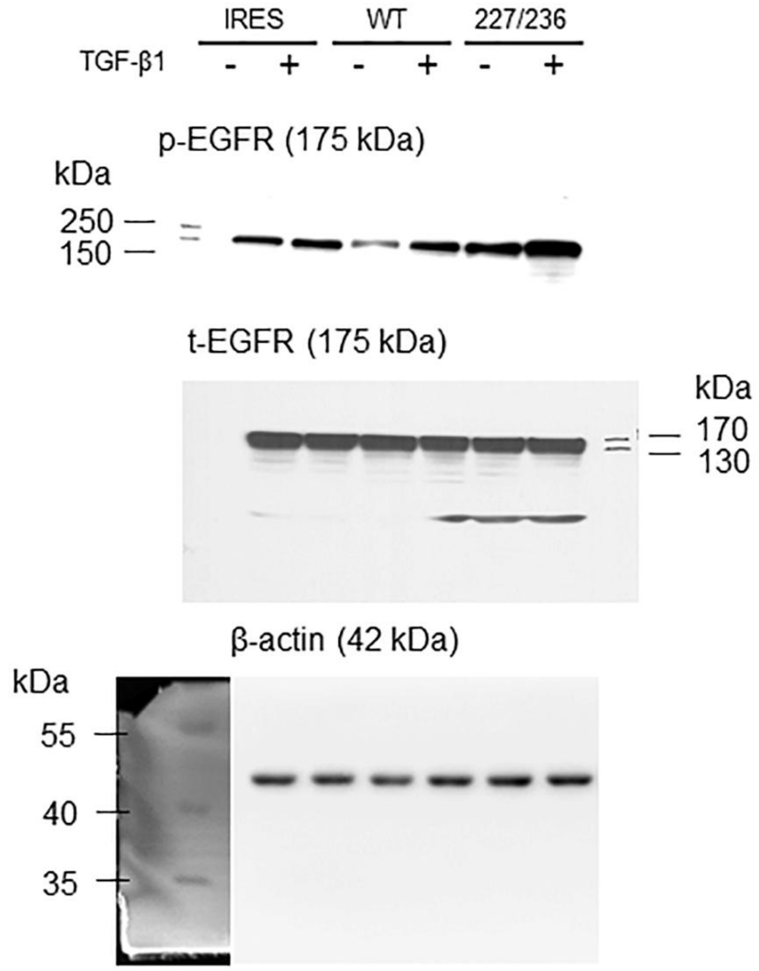

Fig. S6. Full length immunoblots of EGFR protein level (t-EGFR), phosphorylation of EGFR (p-EGFR) and β-actin in **Fig. 5a**. Stable HSC-2 cells were incubated in P medium containing 0.2% FBS in the presence of vehicle (-) or 10 ng/ml of TGF-β1 (+) for 24 h. EGFR protein level (t-EGFR) and phosphorylation of EGFR (p-EGFR) were detected by western blotting. Same protein samples were run three times and transferred to PVDF membranes. Each membrane was cut into two parts and the membrane parts harboring target protein bands were probes with the corresponding antibodies.
